# Commentary: Dietary supplementation with fermented rapeseed and seaweed modulates parasite infections and gut microbiota in outdoor pigs

**DOI:** 10.3389/fvets.2025.1660042

**Published:** 2025-11-27

**Authors:** Nathkapach Kaewpitoon Rattanapitoon, Natnapa Heebkaew Padchasuwan, Patpicha Arunsan, Nav La, Schawanya Kaewpitoon Rattanapitoon

**Affiliations:** 1 Parasitic Diseases Research, FMC Medical Center of Thailand, Nakhonratchasima, Thailand; 2 Faculty of Public Health, Khon Kaen University, Khon Kaen, Thailand; 3 Faculty of Medicine, Vongcahavalitkul University, Nakhonratchasima, Thailand; 4 Faculty of Pediatric and Medicine, International University, Phnom Penh, Cambodia

**Keywords:** fermented feed additives, *Ascaris suum*, gut microbiota, helminth control, rapeseed, seaweed, immunometabolism, sustainable livestock

We read with great interest the recent article by Bonde et al. (1) titled “Dietary supplementation with fermented rapeseed and seaweed modulates parasite infections and gut microbiota in outdoor pigs” published in *Frontiers in Veterinary Science*. The study represents an important advance in the ongoing quest for sustainable parasite control in pig production systems, highlighting the potential of a bioactive dietary intervention in reducing *Ascaris suum* infections and modulating gut microbiota (GM).

In revisiting these findings, this commentary aims not only to summarize but to critically reinterpret them through the emerging framework of systems parasitology—viewing host–parasite–microbiota interactions as an integrated biological network. This approach underscores that dietary modulation affects not a single process but a coordinated triad of host immunity, microbial ecology, and helminth developmental biology.

## Merits and implications

The reported 45.3% reduction in accumulated fecal egg counts (FEC) for *A. suum* in FRS-fed pigs (SUB2–4) underscores the potential anthelmintic properties of fermented rapeseed–seaweed supplements. This observation aligns with prior *in vitro* findings demonstrating larvicidal effects of *Saccharina latissima* extracts against *A. suum* (2). Importantly, these outcomes were achieved under field conditions, enhancing their translational relevance for farm-level parasite control. Similar field-based studies have emphasized the persistent challenge of helminth reinfection in outdoor systems (3, 4).

Beyond the parasitological outcomes, FRS-induced modulation of GM—particularly the enrichment of *Prevotella* spp.—is biologically meaningful. *Prevotella* contributes to complex carbohydrate metabolism and short-chain fatty acid (SCFA) production, both associated with mucosal barrier integrity and immune tolerance (5). These microbial shifts resemble those observed during the restoration of gut eubiosis in postweaning piglets (6), reinforcing the notion that dietary interventions can stabilize microbial communities and support host immune resilience. In the revised framework, we interpret this microbial shift as a possible driver of immunometabolic signaling, potentially influencing Th2–Treg balance and reducing helminth fecundity through SCFA-mediated cytokine modulation. Such cross-kingdom interactions exemplify the dynamic systems-level feedback central to host–parasite–microbiota crosstalk.

From a translational standpoint, we also reposition FRS as a post-anthelmintic nutritional strategy—a promising complement or alternative in the era of escalating benzimidazole and macrocyclic lactone resistance. This recontextualization situates FRS within the broader One Health paradigm, integrating nutrition, microbiota modulation, and sustainable parasite management.

## Points of consideration and future directions

1. **Batch variability and bioactivity**
The disparity in outcomes between FRS batches 1 and 2—particularly the diminished efficacy observed in SUB1—raises critical questions regarding batch consistency. Variations in fermentation parameters (temperature, pH, microbial inoculum) could influence metabolite composition and bioactivity. We therefore propose biochemical fingerprinting of fermented products using LC-MS or NMR-based metabolomic profiling as a quality-control benchmark. Standardization of phytochemical and microbial features is crucial to ensure reproducible outcomes and facilitate cross-study comparisons.
2. **Mechanistic insights beyond observational trends**
While Bonde et al. linked FRS supplementation to changes in GM and infection rates, the causal mechanisms remain to be elucidated. Future work should combine parasite burden quantification (e.g., worm recovery) with mucosal immunophenotyping, cytokine assays, and transcriptomic profiling to uncover whether FRS acts via inhibition of larval establishment, modulation of host immunity, or disruption of worm fecundity. Integration of multi-omics approaches (metabolomics, metagenomics, transcriptomics) could unravel these interactions and clarify host–microbe–helminth signaling pathways.
3. **Limitations of Serological Interpretation**
Uniform seropositivity for anti-*A. suum* antibodies among all pigs—including those without detectable egg excretion—highlights the limits of serology as a marker of active infection (7). Antibody titers may persist after parasite clearance. We therefore suggest combining serology with coproantigen detection, qPCR, or metagenomic diagnostics to provide a more dynamic and precise picture of infection and exposure.
4. **Trade-offs between parasite control and growth performance**
Although FRS supplementation reduced infection incidence, a modest decrease in daily weight gain was observed. This may reflect altered nutrient partitioning or palatability rather than toxicity. The finding underscores a broader nutritional trade-off in phytogenic interventions, where improved health or immune activation may transiently affect energy balance. Similar patterns have been reported in ineffective *Oesophagostomum* control due to ivermectin resistance (8), reinforcing the need to balance biological efficacy with production outcomes.
5. **Relevance to broader contexts**
While the study was conducted under temperate outdoor conditions, future validation in tropical and intensive farming systems is warranted. The conceptual model proposed by Bonde et al.—and expanded here—offers a platform for assessing bioactive feed interventions under diverse ecological and husbandry conditions. Embedding such strategies within a climate-resilient, One Health framework will enhance their applicability across global livestock sectors.

## Conclusion

Bonde et al. (1) have provided a valuable proof-of-concept that fermented rapeseed–seaweed supplementation can beneficially modulate both parasitological and microbiological parameters in pigs under natural exposure. Building upon their work, this commentary advances a systems-level reinterpretation, connecting nutritional bioactivity, microbial ecology, and host immunometabolism into a cohesive model of sustainable parasite control. We advocate for continued efforts toward fermentation standardization, mechanistic validation using multi-omics tools, and cross-system translation of this nutritional innovation. As an emerging post-anthelmintic feed approach, FRS represents a tangible step toward integrative, microbiota-informed solutions for sustainable animal health management.
